# Modulation of Mind Wandering Using Monaural Beat Stimulation in Subjects With High Trait-Level Mind Wandering

**DOI:** 10.3389/fpsyg.2022.815442

**Published:** 2022-06-13

**Authors:** Leila Chaieb, Sofie Krakau, Thomas P. Reber, Juergen Fell

**Affiliations:** ^1^Department of Epileptology, University Hospital Bonn, Bonn, Germany; ^2^Faculty of Psychology, UniDistance Suisse, Brig, Switzerland

**Keywords:** mind wandering, monaural beat, auditory beat stimulation, sustained attention to response task, mind wandering questionnaire, meta-awareness, temporal orientation

## Abstract

Mind wandering (MW) refers to a state when attention shifts from the task at hand or current situation toward thoughts, feelings, and imaginations. This state is often accompanied by a decline in mood, and patients suffering from major depression exhibit more perseverative MW. Hence, although the directionality of the relationship between mood and MW is still under investigation, it may be useful to explore possible avenues to reduce MW. In an earlier pilot study, we investigated MW during auditory beat stimulation in healthy subjects using thought-probes during a sustained attention to response task (SART). We found evidence for reduced MW during monaural 5 Hz beats compared to silence, sine tones, and binaural 5 Hz beats. Moreover, the data tentatively suggested that this reduction was particularly pronounced in subjects with high levels of MW during silence. In the current study, we therefore asked whether MW can be reduced by monaural theta beats in subjects with high trait-levels of MW, as indicated by an online MW questionnaire. Preselected subjects performed a SART task with thought-probes assessing the propensity to mind wander, meta-awareness, and the temporal orientation of MW. Stimulation conditions comprised monaural theta beats, as well as silence (headphones on), and sine tones as control conditions. Our main hypothesis stating that the propensity to mind wander during monaural theta beats is reduced compared to both control conditions was only partly confirmed. Indeed, MW was significantly diminished during exposure to the theta beats compared to sine tones. However, reduced MW during theta beats versus silence was only observed in a subgroup using stricter inclusion criteria. Considering possible reasons for this outcome, our data suggest that the preselection procedure was suboptimal and that beat effects are modulated by the individual responses to auditory stimulation in general.

## Introduction

The term mind wandering (MW) commonly refers to the drift of attention inward toward thoughts, feelings, and imaginations which are not related to the task at hand or current situation (Cohen et al., 1956; Smallwood and Schooler, 2015). Reduced mood has been shown to be followed by more frequent episodes of MW (Smallwood et al., 2009). Conversely, a decline in mood has also been reported to follow not only unpleasant, but also neutral MW (Killingsworth and Gilbert, 2010). Broadly speaking, this vicious cycle may contribute to the emergence of depressive disorders (Fell, 2012; Chaieb et al., 2022). Further to this, patients suffering from major depression exhibit more perseverative MW, in particular, repetitive thought patterns concerning negative issues from the past and worries about the future (Ottaviani et al., 2015; Hoffmann et al., 2016). Hence, methods allowing to diminish MW or to shift its temporal orientation toward the present would be desirable.

One current approach that aims at reducing MW is to try to enhance mindfulness, for instance, in the context of mindfulness-based cognitive therapy practices (Segal et al., 2012). The central tenet of mindfulness is to focus one’s full attention on what is experienced in the present moment without any form of judgment. However, mindfulness training is demanding, requires disciplined practice, which is often beyond the capabilities of many patients, and can produce adverse effects (e.g., Farias et al., 2016; Groves, 2016). Non-invasive interventional methods such as transcranial electric brain stimulation may provide an alternative option. However, existing studies exhibit considerable methodological differences and to some extent report contradictory findings (Chaieb et al., 2019).

A new, upcoming non-invasive brain stimulation method is auditory beat stimulation. A simple implementation of this technique is to apply sine tones with slightly different frequencies. For instance, two sine tones with frequencies of 217 and 223 Hz would generate a beat at 6 Hz, i.e., in the theta range. The tones are either superposed resulting in amplitude modulated signals (monaural beats), or they are applied separately to each ear (binaural beats). Both application types create a beat sensation, the former a result of physical properties, the latter due to the activity of phase-sensitive neurons in the brain stem. Auditory beats have been shown to alter EEG dynamics (Schwarz and Taylor, 2005; Becher et al., 2015), as well as to modulate memory performance (Chaieb et al., 2015; Garcia-Argibay et al., 2019). More specifically, we found a reduction of intracranial EEG power and phase synchronization in rhinal cortex and hippocampus due to 5 Hz monaural beat versus sine wave stimulation, which was not observed for 5 Hz binaural beats (Becher et al., 2015). Since the hippocampus has been shown to play a major role in MW (e.g., Ellamil et al., 2016; O’Callaghan et al., 2019), we would expect that a reduction of hippocampal EEG power and phase synchronization may decrease MW. Based on these findings, we therefore formed the hypothesis that a reduction of MW may result from monaural beat stimulation in the theta range.

In a recent pilot study (Chaieb et al., 2020; sample size *N* = 40), we investigated MW during auditory beat stimulation in healthy subjects using thought-probes during a sustained attention to response task (SART; Robertson et al., 1997). Stimulation conditions comprised of monaural and binaural beats at 5 and 40 Hz, as well as a sine tone, and silence (headphones on) as control conditions (within-subjects manipulation). Indeed, we found evidence for decreased MW during monaural 5 Hz beats compared to the silence condition, the sine tone, and the binaural 5 Hz beat stimulation, which was in line with our initial hypothesis (average differences: −11.4%, −5.9%, and −10.2%). However, we observed no overall modulation of MW when evaluated across all conditions. Interestingly, the magnitudes of the reductions versus silence strongly depended on the levels of MW during silence (*r* = −0.467; *p* = 0.002). The average decrease of MW during monaural 5 Hz beats versus silence amounted to −23.6% in a subgroup with high MW levels during silence (median-split). However, we cannot exclude that regression to the mean contributed to this finding.

In this current investigation, we therefore tested the hypothesis that MW can be reduced by monaural theta beats in healthy subjects with high trait-levels of MW. Subjects who took part in the study were preselected based on a well-established MW questionnaire (Mrazek et al., 2013). Similar to our previous study, MW was assessed using thought-probes intermittently dispersed during a SART task. These thought-probes addressed the propensity to MW, meta-awareness, and the temporal orientation of MW. In order to render monaural theta beats less monotonous for the participants, the modulation frequency was continuously shifted between 4 Hz and 8 Hz. Again, control conditions consisted of silence (headphones on) and a pure sine tone. Our main hypothesis was that the propensity to mind wander during monaural theta beats would be reduced compared to both control conditions. Furthermore, we had no specific hypothesis regarding the temporal orientation of MW and expected no dependence of meta-awareness on stimulation conditions.

## Materials and Methods

### Participants

In total, 107 subjects (mean age ± SEM: 26.2 ± 0.6; 80 female) took part in an online survey, evaluating levels of MW using a well-established questionnaire (Mrazek et al., 2013). Based on overall MW scores, we selected subjects with scores above or equal to the median (median score = 17). These subjects with presupposed high trait-level MW were invited to participate in the experimental part of the study, and 34 subjects accepted this invitation to do so (age 25.7 ± 0.8, 23 female). In addition to the analyses of data from this group, also a subgroup with tighter selection criteria (scores ≥19) was subjected to exploratory analyses (see below).

### Recruitment Procedure

Subject recruitment took place *via* advertisements on social media, posters, and a website created for the purpose of the study, providing all necessary information. Each participant received monetary compensation for their attendance (20 Euro/h; approx. 2.5 h). The online survey was created and hosted using the LamaPoll questionnaire platform.^1^ Both the website and online survey were closed after the recruitment period of 3 months had expired. All subjects gave informed consent for their participation in the online survey and for the subsequent experimental procedure, which was conducted in line with COVID-19 protection guidelines, as per the German Ministry of Health. The study was approved by the Ethics Committee of the Medical Faculty of the University of Bonn, and all procedures were in accordance with the Declaration of Helsinki.

### Self-Rating Mind Wandering Scale

All participants of the survey performed an online version of the mind-wandering questionnaire (Mrazek et al., 2013). This frequently used self-rating questionnaire is comprised of five questions, which have to be answered using a six-point Likert scale.

### Experimental Paradigm

**Figure 1:**
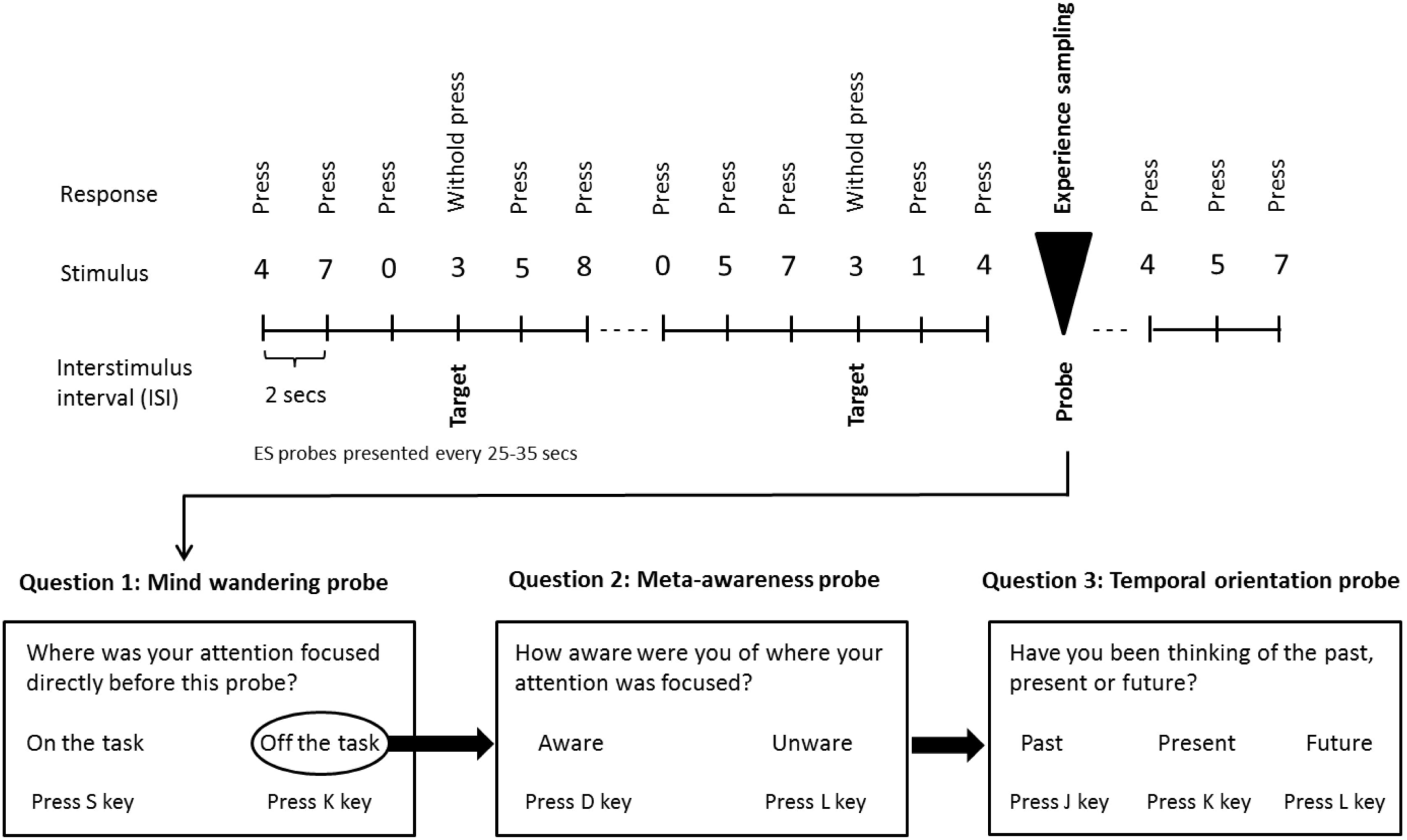
Schematic illustration of the sustained attention to response task (SART) with embedded experience sampling probes. Subjects are instructed to respond with a button press when a non-target digit appears on the screen, with the exception of the target digit (3) for which they were instructed to withhold the button press. Subjects were also asked to respond to the embedded intermittent experience sampling probes when they appeared onscreen [probe 1: “Where was your attention focused immediately before the probe appeared?” (possible responses: “on task” or “off task”). Whenever participants indicated being “off-task,” a second probe assessed meta-awareness: “Were you aware that your attention was off-task?” (possible responses: “yes” or “no”). A third probe inquired as to the temporal orientation of MW: “Have you been thinking of the past, present or future?” (possible responses: “past,” “present,” and “future”)]. Participants were asked to respond as quickly and as accurately as possible.

Selected participants, showing a purportedly greater tendency to mind wander, came to the Department of Epileptology, University Hospital Bonn and performed a variant of the sustained attention to response task (SART; Robertson et al., 1997). They were asked to follow a continuous stream of digits onscreen and to press the space bar whenever a non-target number (0–2 and 4–9) occurred (see Figure 1). They were further instructed to withhold the bar-press whenever the target number (3) appeared onscreen. Stimuli were presented until a response was detected, or for a maximum duration of 2 s, with the inter-stimulus interval being 2 s. Participants were instructed to respond as quickly and as accurately as possible. Each participant performed three runs of the SART. Each run had a duration of approximately 35 min (i.e., responses to 60 experience sampling probes were acquired per run; see below), and during each run, a different auditory beat stimulation condition was administered (within-subjects manipulation). The order of runs was counterbalanced across subjects. Between runs, there were short breaks of about 10 min duration each.

### Experience Sampling

To examine the propensity to mind wander, meta-awareness, and the temporal orientation of MW, experience sampling probes were embedded intermittently within the SART digit stream (see Figure 1). The first probe addressed the subject’s focus of attention immediately before appearance of the probe: “Where was your attention focused immediately before the probe appeared?” (possible responses: “on task” or “off task”). Whenever participants indicated being “off-task,” a second probe examined their meta-awareness: “Were you aware that your attention was off-task?” (possible responses: “yes” or “no”). Finally, a third probe inquired as to the temporal orientation of MW: “Have you been thinking of the past, present or future?” (possible responses: “past,” “present,” and “future”). Inter-probe intervals varied between 25 s and 35 s (Schubert et al., 2020) and the typical number of probes (1-fold or 3-fold) per run was 60.

### Auditory Beat Stimulation

To examine the effects of monaural beat stimulation, three different stimulation conditions were applied as: (i) a “headphones only” condition (control 1), (ii) a 220 Hz sine tone (control 2; frequency identical to the carrier frequency of the monaural beat), and (iii) a 4–8 Hz monaural beat. The order of stimulation conditions (across runs) was counterbalanced across subjects. The monaural beat used for the current study was constructed as follows: two sine waves were superposed, whose frequencies fell/rose within 30 s from 218 to 216 Hz (sine wave 1), and from 222 to 224 Hz (sine wave 2), then rose/fell within 30 s back to 218 Hz (sine wave 1), and 222 Hz (sine wave 2), and so forth. This procedure resulted in a monaural beat with a carrier frequency of 220 Hz and a modulation frequency which continuously shifted between 4 Hz and 8 Hz. All auditory stimuli were played using over-ear headphones and with a sound pressure level (SPL) of 60 dB. The SPL was adjusted to 55 dB when participants found the preset volume uncomfortable (occurred only in 2/34 cases). Auditory beats were played using the Windows Media player application and were created using Tone Generator (NCH software, Canberra, Australia).

### Statistical Analyses

Based on the thought-probes, the following MW measures were analyzed as: (i) propensity to mind wander (i.e., proportion of “off-task” responses to first probe question); (ii) ratio of meta-awareness (denominator: all “off task” responses); and (iii) ratios of present/past/future orientation (denominator: all “off task” responses). Additionally, based on the SART, the following behavioral measures were evaluated as: (i) accuracy of responses to non-targets; (ii) accuracy of responses to targets; and (iii) reaction times after non-targets. Statistical comparisons between stimulation conditions were performed using paired Wilcoxon signed-rank tests. In addition to *p*-values, the test statistic *z*, as well as effect sizes *r* (*r* = |*z*|/√*n*; Rosenthal, 1994) are reported. The following two-pronged main hypothesis was tested (one-tailed Wilcoxon): reduced propensity to mind wander during monaural theta beats compared to (i) silence (headphones on) and (ii) the sine tone. All additional tests were exploratory in nature (two-tailed Wilcoxon).

Moreover, Spearman correlations between experience sampling-based MW propensities and questionnaire-based MW scores, as well as behavioral SART measures were evaluated [significance threshold (two-tailed): *p* = 0.05].

Finally, in order to explore the effect of tighter inclusion criteria, we tested our two-pronged hypothesis (one-tailed Wilcoxon) in a subgroup of participants with higher trait-level MW scores (*n* = 20). In this subgroup, questionnaire-based MW scores were larger than the average value (18.86, i.e., scores ≥19) for the group of 663 subjects investigated in the original study by Mrazek et al. (2013).

## Results

### Differences Between Stimulation Conditions

**Figure 2:**
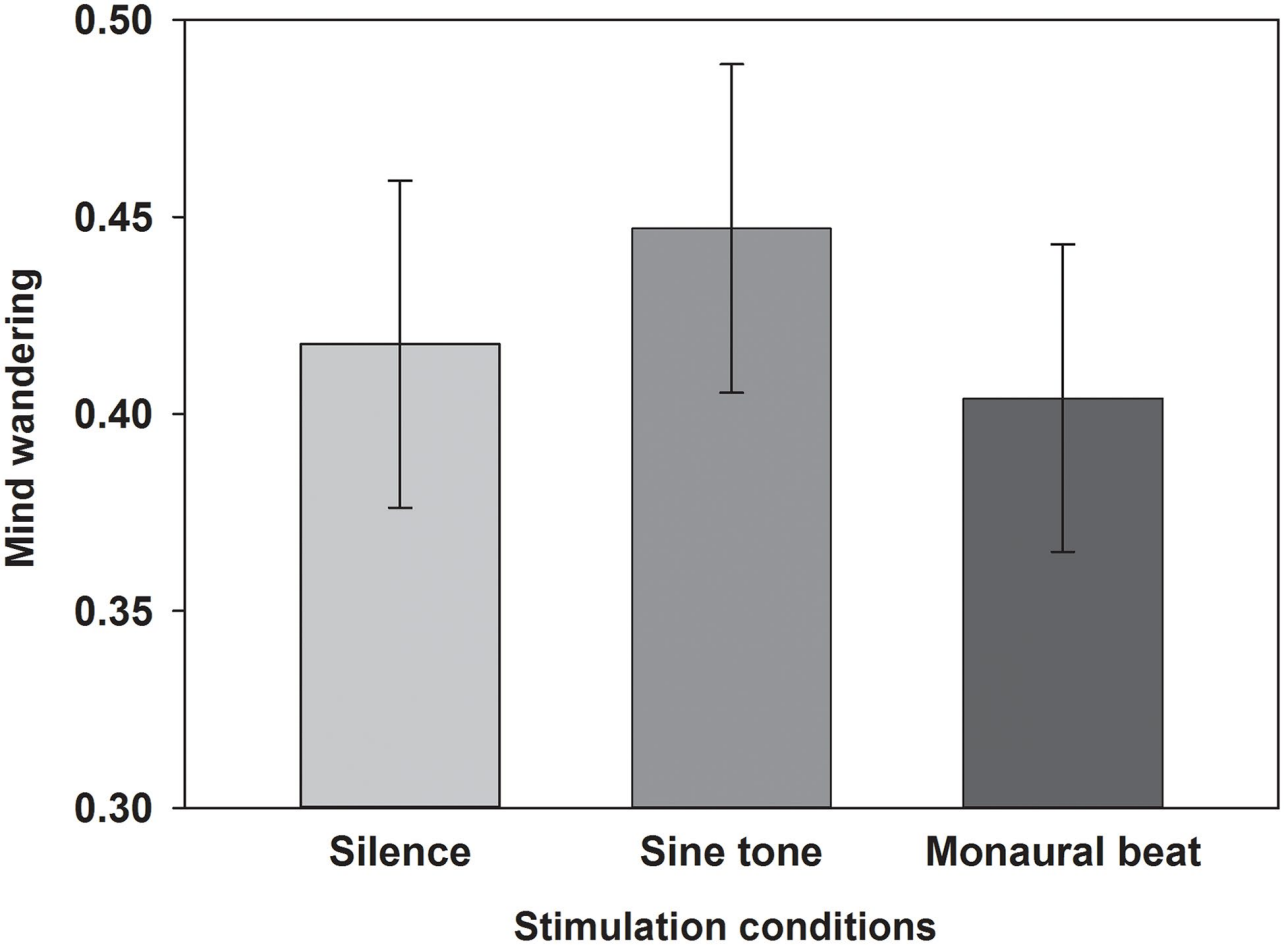
Propensity to mind wander. The average propensity to mind wander shown across subjects for the three stimulation conditions. Mean and SEM are depicted.

**Figure 3:**
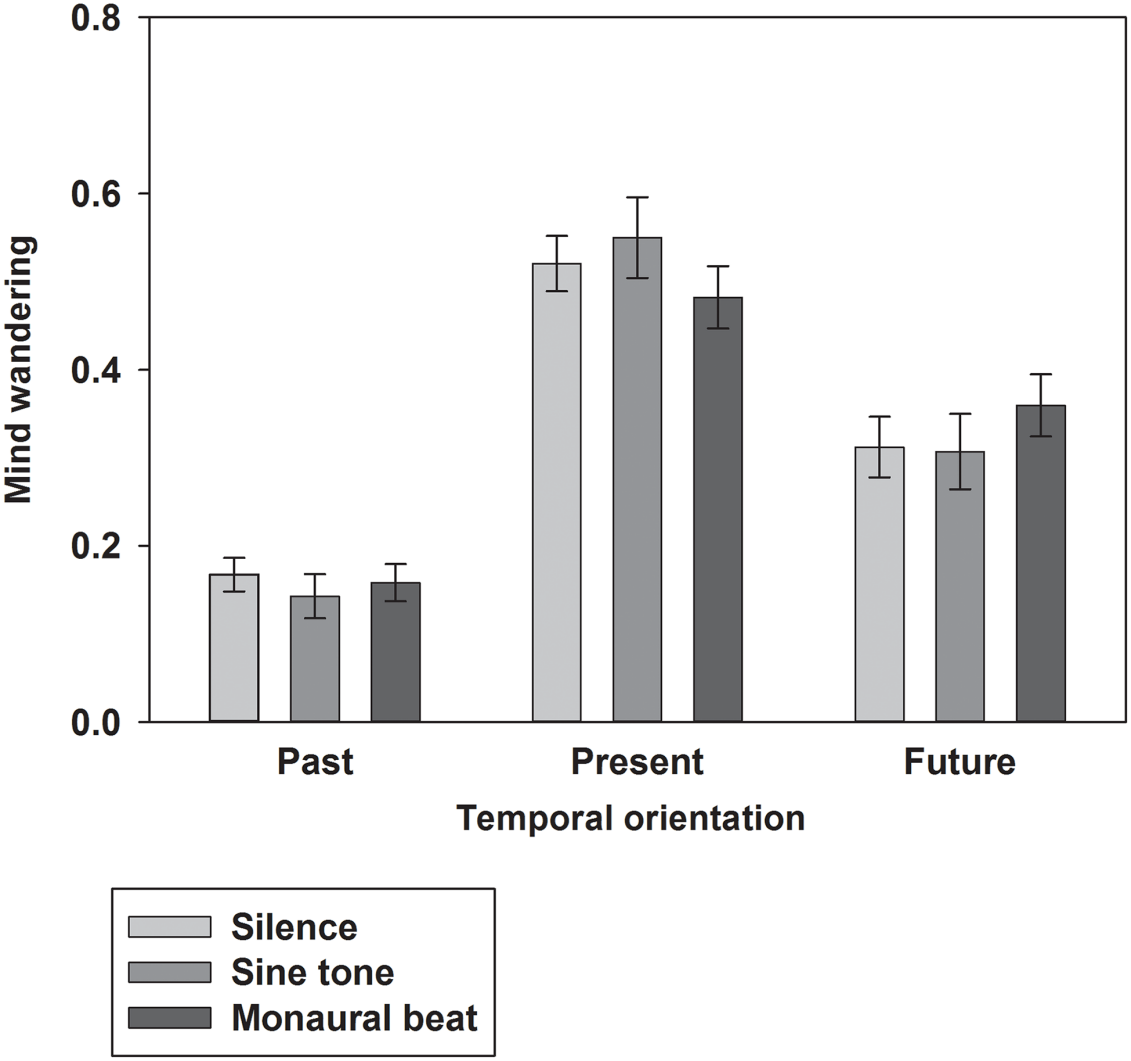
Temporal orientation of mind wandering. The average ratios of past-, present-, and future-oriented MW shown across subjects for the three stimulation conditions. Mean and SEM are depicted.

First, we tested our two-pronged main hypothesis in the subjects who showed high trait-level mind wandering (*n* = 34; MW scores ≥17; mean score ± SEM: 19.7 ± 0.4). Indeed, the propensity to mind wander was reduced during monaural beats compared to the sine tone (average ± SEM, monaural beat: 0.404 ± 0.039; sine tone: 0.447 ± 0.042; one-tailed Wilcoxon test, *z* = −2.161, *p* = 0.016; and effect size *r* = 0.37; Figure 2). However, we did not find evidence for decreased MW during monaural beats compared to silence (silence: 0.418 ± 0.042; *z* = −0.633, *p* = 0.264; *r* = 0.11). The additional exploratory tests (two-tailed Wilcoxon) revealed more future-oriented MW (*z* = −2.135, *p* = 0.033; *r* = 0.37), as well as marginally non-significant reduced present-oriented MW (*z* = −1.939, *p* = 0.053; *r* = 0.33) during monaural beats versus sine tone (Figure 3). No statistically significant differences between stimulation conditions were detected for meta-awareness and the behavioral SART measures (Table 1).

**Table 1:** Summary of percentages of meta-awareness and SART behavioral measures.

Stimulation condition	Percentages of meta-awareness		SART behavioral measures		
	Aware (%)	Unaware (%)	Correct responses to non-targets (%)	Correct responses to targets (%)	Reaction time (ms)
Silence (headphones only)	47.85 (5.37)	52.15 (5.37)	99.02 (0.23)	76.33 (3.11)	415.68 (10.82)
Sine tone	43.98 (5.28)	56.02 (5.28)	98.84 (0.39)	75.16 (3.71)	413.36 (10.49)
Monaural beat	43.73 (5.07)	56.27 (5.07)	98.88 (0.35)	75.34 (2.82)	405.25 (8.73)

*Average values and SEM for the meta-awareness probe and the SART behavioral measures, for each stimulation condition across all participants, are depicted*.

### Relation Between MW Changes During Monaural Beats vs. Silence and Questionnaire-Based MW Scores

**Figure 4:**
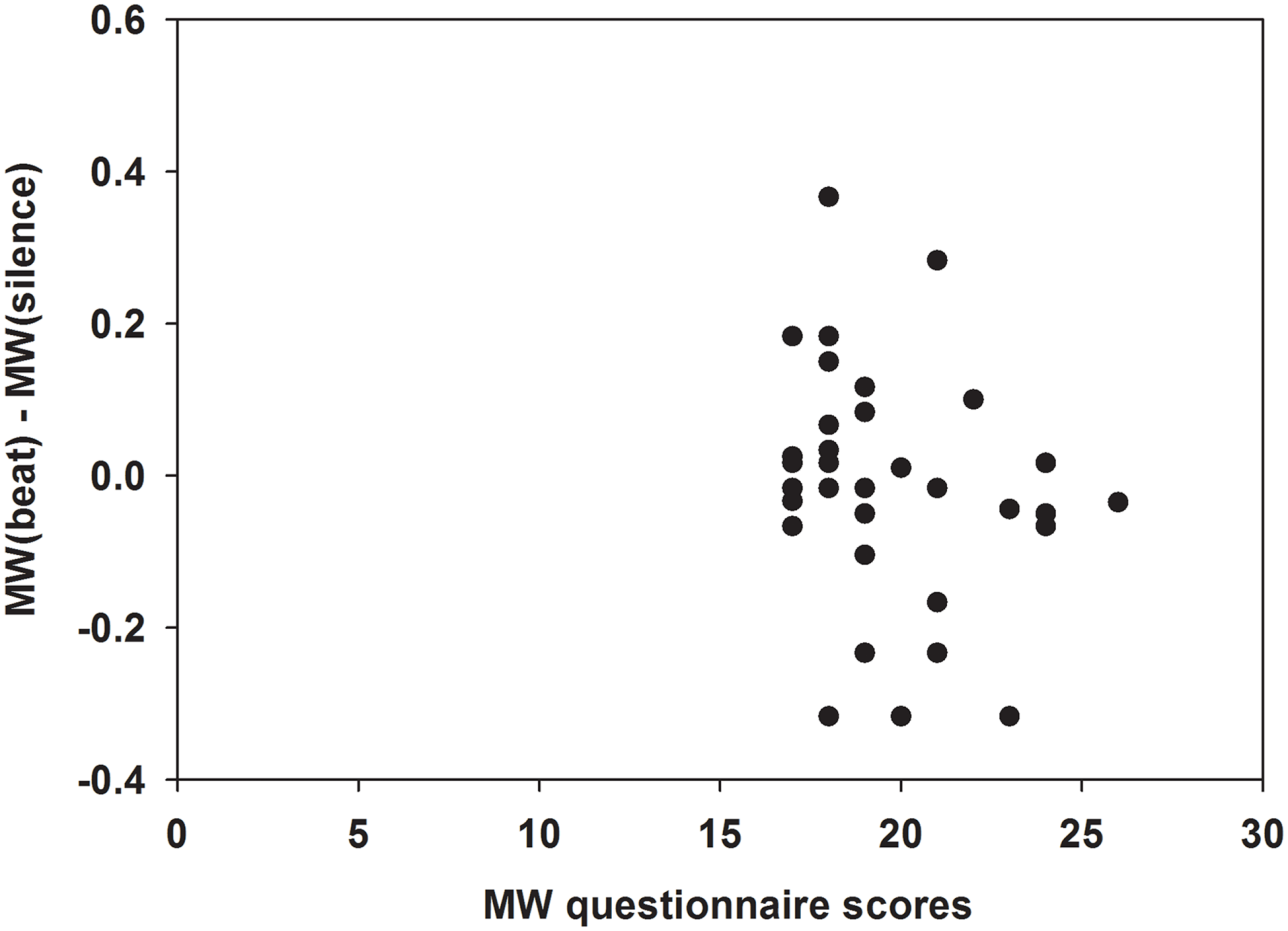
Dependence of differences in MW propensities during monaural beats versus silence on MW scores. Differences in MW propensities during monaural beats versus silence [i.e., MW(beat)—MW(silence)] in the overall group (*n* = 34). Differences in MW propensities were negatively correlated with questionnaire-based MW scores (Spearman’s correlation: *ρ* = −0.322; *p* = 0.063).

There was a trend for a negative correlation between differences of MW propensities during monaural beats versus silence [i.e., MW(beat)—MW(silence)] and questionnaire-based MW scores (*ρ* = −0.322; *p* = 0.063; Figure 4).

### Correlations Between the MW and SART Measures

Experience sampling-based MW propensities were significantly correlated with questionnaire-based MW scores during silence (Spearman’s *ρ* = 0.398; *p* = 0.020), but not during the sine tone condition (*ρ* = 0.244; *p* = 0.164) or the monaural beat (*ρ* = 0.257; *p* = 0.142; see also Table 2). However, correlations between experience sampling-based MW propensities and questionnaire-based MW scores did not significantly change between conditions (silence/sine: *z* = 1.224, silence/beat: *z* = 1.149, sine/beat: *z* = −0.099; each *p* > 0.1). Moreover, MW propensities were negatively correlated with accuracies of responses to non-targets during the sine tone (*ρ* = −0.445; *p* = 0.008) and during monaural beats (*ρ* = −0.436; *p* = 0.010), and we observed a trend for a negative correlation during silence (*ρ* = −0.305; *p* = 0.079). Furthermore, MW propensities were significantly correlated with reaction times during the monaural beats (*ρ* = 0.508; *p* = 0.002), but not during silence (*ρ* = 0.097; *p* = 0.584) or the sine tone (*ρ* = 0.073; *p* = 0.683).

**Table 2:** Summary table of Spearman’s correlations with MW propensities.

Experimental condition	Silence (headphones only)	Sine tone	Monaural beat
Measures			
MW scores (questionnaire-based)	0.398 (*p* = 0.020)	0.244 (*p* = 0.164)	0.257 (*p* = 0.142)
Accuracy for non-targets	−0.305 (*p* = 0.079)	−0.445 (*p* = 0.008)	−0.436 (*p* = 0.010)
Accuracy for targets	n.s.	n.s.	n.s.
Reaction times	0.097 (*p* = 0.584)	0.073 (*p* = 0.683)	0.508 (*p* = 0.002)

*Spearman’s correlations of MW scores and SART measures with MW propensities for each stimulation condition. Significance values (two-tailed) are depicted in brackets*.

### Subgroup With MW Scores ≥19

**Figure 5:**
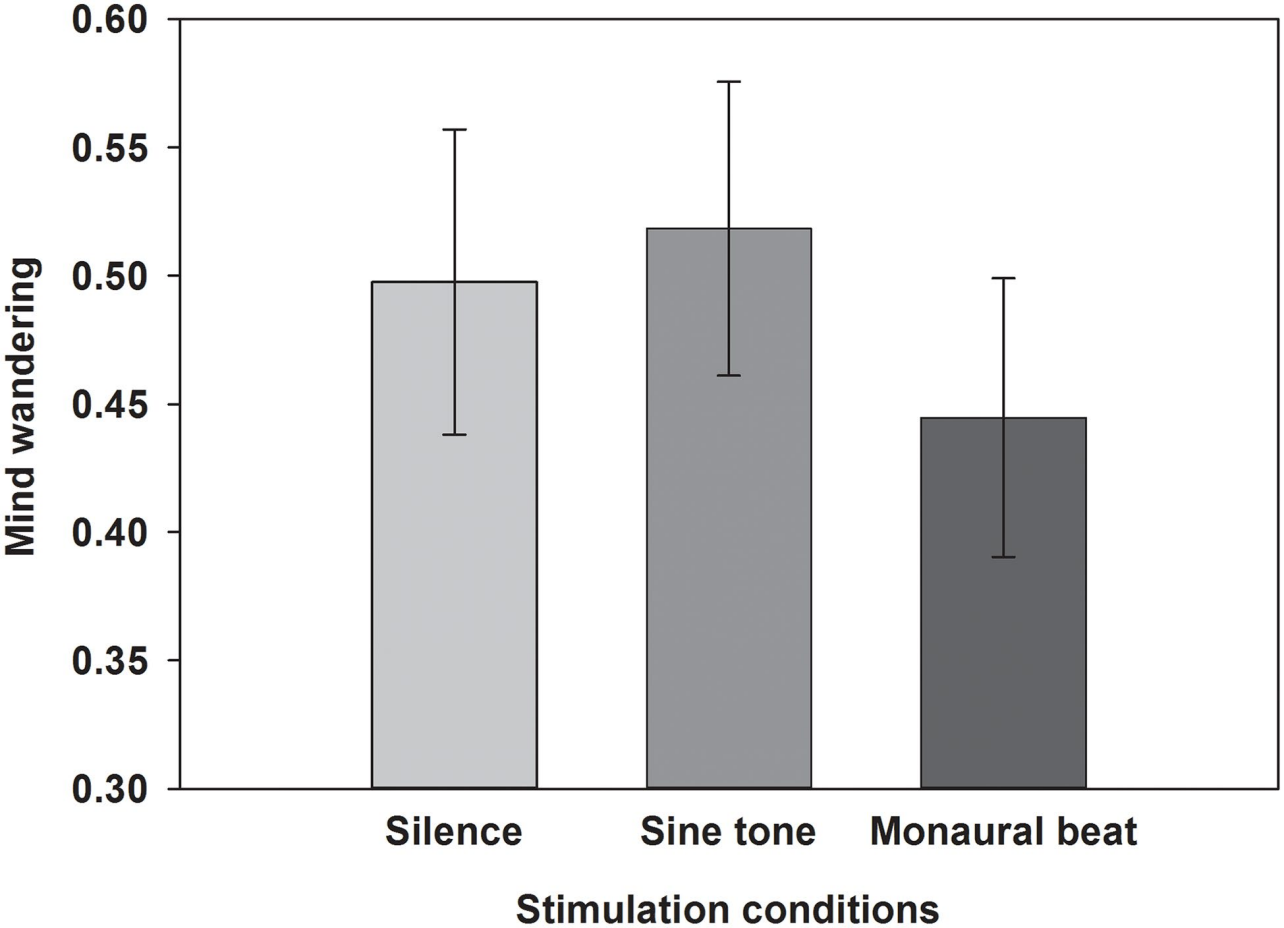
Propensity to mind wander in subgroup with high MW questionnaire scores. The average propensity to mind wander shown across subjects in a subgroup with high MW questionnaire scores (≥19) for the three stimulation conditions. Mean and SEM are depicted.

Finally, we tested our two-pronged main hypothesis in a subgroup (*n* = 20) using tighter inclusion criteria (MW scores ≥ 19; mean score ± SEM: 21.2 ± 0.4). In this subgroup, there was not only a significant difference of MW propensities during monaural beats versus sine tone (average ± SEM, monaural beat: 0.445 ± 0.042; sine tone: 0.518 ± 0.044; one-tailed Wilcoxon test: *z* = −2.114, *p* = 0.018; *r* = 0.47), but also a significant reduction during monaural beats versus silence (silence: 0.498 ± 0.046; *z* = −1.756, *p* = 0.040; *r* = 0.39; Figure 5).

## Discussion

In this study, we investigated the effects of monaural beat stimulation in the theta range on MW propensities in subjects with high questionnaire-based MW scores (≥ 17). Similar to our previous findings (Chaieb et al., 2020), we observed significantly reduced MW during monaural beats compared to the sine tone (average difference, present study: −9.6%; previous study: −5.9%). However, we did not observe general evidence for significantly reduced MW during monaural theta beats compared to silence (average difference: −3.3%; previous study: −11.4%). Such evidence was only found after performing an additional analysis in a subgroup with stricter inclusion criteria (MW scores ≥ 19; average difference: −10.6%). In line with this outcome, differences of MW during monaural beats versus silence were negatively correlated with questionnaire-based scores. Therefore, it is possible that reduced MW during monaural beats, compared to both the sine tone and silence conditions, is only evident in subjects with higher trait-level scores than those included in the present study. Moreover, we cannot exclude that beat stimulation effects are stronger for the monaural 5 Hz beats applied in the previous study, than for the monaural beats with frequencies varying between 4 and 8 Hz used in the present study.

Interestingly, average MW propensities during silence were numerically even lower in the present study compared to the previous one (present: 0.418; previous: 0.429; Mann–Whitney U-test: *z* = 0.0054, *p* = 0.99). This suggests that our preselection procedure based on a questionnaire (Mrazek et al., 2013) was not effective in the sense that it did not reliably identify subjects showing higher experience sampling-based MW propensities. In previous studies, moderate correlations between scores of the MW questionnaire (Mrazek et al., 2013) and MW data based on experience sampling during the SART were reported (Kawagoe et al., 2020; Krakau et al., 2020). Generally, it was found that experience sampling-based MW reports not only reflect MW traits, but also individual tendencies to mind wander during specific tasks (Rummel et al., 2021). Moreover, questionnaire-based MW data most likely to a larger extent depend on self-reflection and insight than experience sampling-based reports do (Hurlburt et al., 2022). Thus, one may expect that questionnaire-based MW scores and MW propensities measured during the SART do not perfectly map onto one another. However, it remains unclear why our preselection procedure was not at least in part effective. Possibly, it cannot be excluded that subjects did not fill out the online questionnaire in an entirely honest manner, and in some sense may have attempted to anticipate what the aims of the study were. Although we did withhold the information, that those participants with a high score would be selected for the experimental part of the study, subjects may have predicted this.

Another unexpected outcome was the proportion of average MW propensities during the two control conditions. Average MW propensities during the sine tone condition were numerically larger than during silence, in contrast to the data from our previous study (average differences: present: +7.0%; previous: −6.9%). One may wonder whether some subjects tend to respond with increased MW to any type of auditory stimulation. Indeed, differences of MW propensities during the sine tone versus silence and during the monaural theta beats versus silence had more often the same sign than expected by chance, in both the present and in the previous study [present: 23 of 34 (68%); previous: 26 of 40 (65%); and each *p* < 0.05 (binomial tests)]. This suggests that the attempted reduction of MW using beat stimulation depends on the general response to auditory stimulation and that this approach may not be applicable to every subject.

Furthermore, a potential reason for the divergence in findings between the present study and those of the previous study could be that the current study was conducted during the first COVID-19 lockdown, and in accordance with the regulations of the German Ministry of Health (this included social distancing, the wearing of face masks, and regular disinfection of the surrounding area). However, the previous study (Chaieb et al., 2020) was performed under normal (pre-pandemic) conditions. Two recent studies have reported outcomes related to the COVID-19 pandemic impacting upon processes linked to MW (Lopez et al., 2021; Brosowsky et al., 2022). Lopez and colleagues describe that “worries related to the pandemic produced psychological maladjustment and cognitive discomforts, such as cognitive failures and ruminative thinking, related to mind wandering” (Lopez et al., 2021). The second study observed that the strictures of the enforced lockdown exerted a negative impact on mood and wellbeing of individuals who showed higher levels of boredom proneness. This negative effect was attenuated by the pursuing of creative tasks (Brosowsky et al., 2022). These studies indicate that the pandemic situation may have somewhat influenced processes related to MW and the general feeling of wellbeing, and in turn, might have affected the MW changes in response to auditory stimulation in general, and beat stimulation, in particular.

Concerning the impact of auditory beat stimulation on the temporal orientation of MW, exploratory statistical comparisons revealed a larger ratio of future-oriented MW and a trend for a smaller ratio of present-oriented MW during monaural beats versus the sine tone. These results should be regarded with caution, since these tests were not based on any specific hypotheses. Mind wandering in depression is often related to negative issues emanating from the past and worries concerning the future (Ottaviani et al., 2015; Hoffmann et al., 2016). Hence, one may speculate that particularly targeting the reduction of past- and future-related MW may be desirable from a clinical point of view. Our provisional findings in healthy subjects appear not to be in line with this ambition.

Finally, our data do support the notion of an influence of MW on performance in the SART task. MW propensities were negatively correlated with accuracies of responses to non-targets and positively correlated with reaction times, in accordance with findings of previous studies (e.g., McVay and Kane, 2012; Leszczynski et al., 2017). However, the latter correlation was only observed during the application of monaural beats. Speculatively speaking, the increased perceptual load due to beat stimulation may have amplified the impact of MW on reaction times.

To summarize, our hypothesis that the propensity to MW would be reduced during monaural theta beats versus both control conditions in subjects with high trait-level MW was only partly confirmed. We observed significantly diminished MW during exposure to the theta beats compared to sine tones. However, reduced MW during theta beats versus silence was only detected in a subgroup using stricter inclusion criteria. One possible reason for this outcome may be that our preselection procedure, based on an online questionnaire, was not optimal. Moreover, the individual responses to auditory stimulation, in general, may modulate the impact of beat stimulation on MW. Further studies are needed to unravel these and other factors underlying alterations of MW during beat stimulation.

## Data Availability Statement

The raw data supporting the conclusions of this article will be made available by the authors, without undue reservation.

## Ethics Statement

The studies involving human participants were reviewed and approved by the Ethics Committee of the Medical Faculty of the University of Bonn, and all procedures were in accordance with the Declaration of Helsinki. The patients/participants provided their written informed consent to participate in this study.

## Author Contributions

LC, SK, and JF conceived and designed the study and performed the analysis of the data. SK and LC collected the data. JF wrote the first draft of the manuscript. LC, SK, and TR contributed to sections to the manuscript. LC, TR, and JF revised the manuscript. All authors contributed to the article and approved the submitted version.

## Conflict of Interest

The authors declare that the research was conducted in the absence of any commercial or financial relationships that could be construed as a potential conflict of interest.

## Publisher’s Note

All claims expressed in this article are solely those of the authors and do not necessarily represent those of their affiliated organizations, or those of the publisher, the editors and the reviewers. Any product that may be evaluated in this article, or claim that may be made by its manufacturer, is not guaranteed or endorsed by the publisher.

## Footnotes

^1^ https://www.lamapoll.de/
